# The Comparative Toxicogenomics Database's 10th year anniversary: update 2015

**DOI:** 10.1093/nar/gku935

**Published:** 2014-10-17

**Authors:** Allan Peter Davis, Cynthia J. Grondin, Kelley Lennon-Hopkins, Cynthia Saraceni-Richards, Daniela Sciaky, Benjamin L. King, Thomas C. Wiegers, Carolyn J. Mattingly

**Affiliations:** 1 Department of Biological Sciences, North Carolina State University, Raleigh, NC 27695-7617, USA; 2 Department of Bioinformatics, The Mount Desert Island Biological Laboratory, Salisbury Cove, ME 04672, USA

## Abstract

Ten years ago, the Comparative Toxicogenomics Database (CTD; http://ctdbase.org/) was developed out of a need to formalize, harmonize and centralize the information on numerous genes and proteins responding to environmental toxic agents across diverse species. CTD's initial approach was to facilitate comparisons of nucleotide and protein sequences of toxicologically significant genes by curating these sequences and electronically annotating them with chemical terms from their associated references. Since then, however, CTD has vastly expanded its scope to robustly represent a triad of chemical–gene, chemical–disease and gene–disease interactions that are manually curated from the scientific literature by professional biocurators using controlled vocabularies, ontologies and structured notation. Today, CTD includes 24 million toxicogenomic connections relating chemicals/drugs, genes/proteins, diseases, taxa, phenotypes, Gene Ontology annotations, pathways and interaction modules. In this 10th year anniversary update, we outline the evolution of CTD, including our increased data content, new ‘Pathway View’ visualization tool, enhanced curation practices, pilot chemical–phenotype results and impending exposure data set. The prototype database originally described in our first report has transformed into a sophisticated resource used actively today to help scientists develop and test hypotheses about the etiologies of environmentally influenced diseases.

## CTD's 10TH YEAR ANNIVERSARY

On 12 November 2004, the Comparative Toxicogenomics Database (CTD; http://ctdbase.org/) was launched on the web (1,2). Over the last decade, CTD has evolved into a premier toxicology resource connecting chemicals, genes/proteins, diseases, taxa, Gene Ontology (GO) annotations and pathways (3–8). Here, we celebrate some of the defining changes, features and enhancements, as well as present our newest updates.

**Figure 1.**
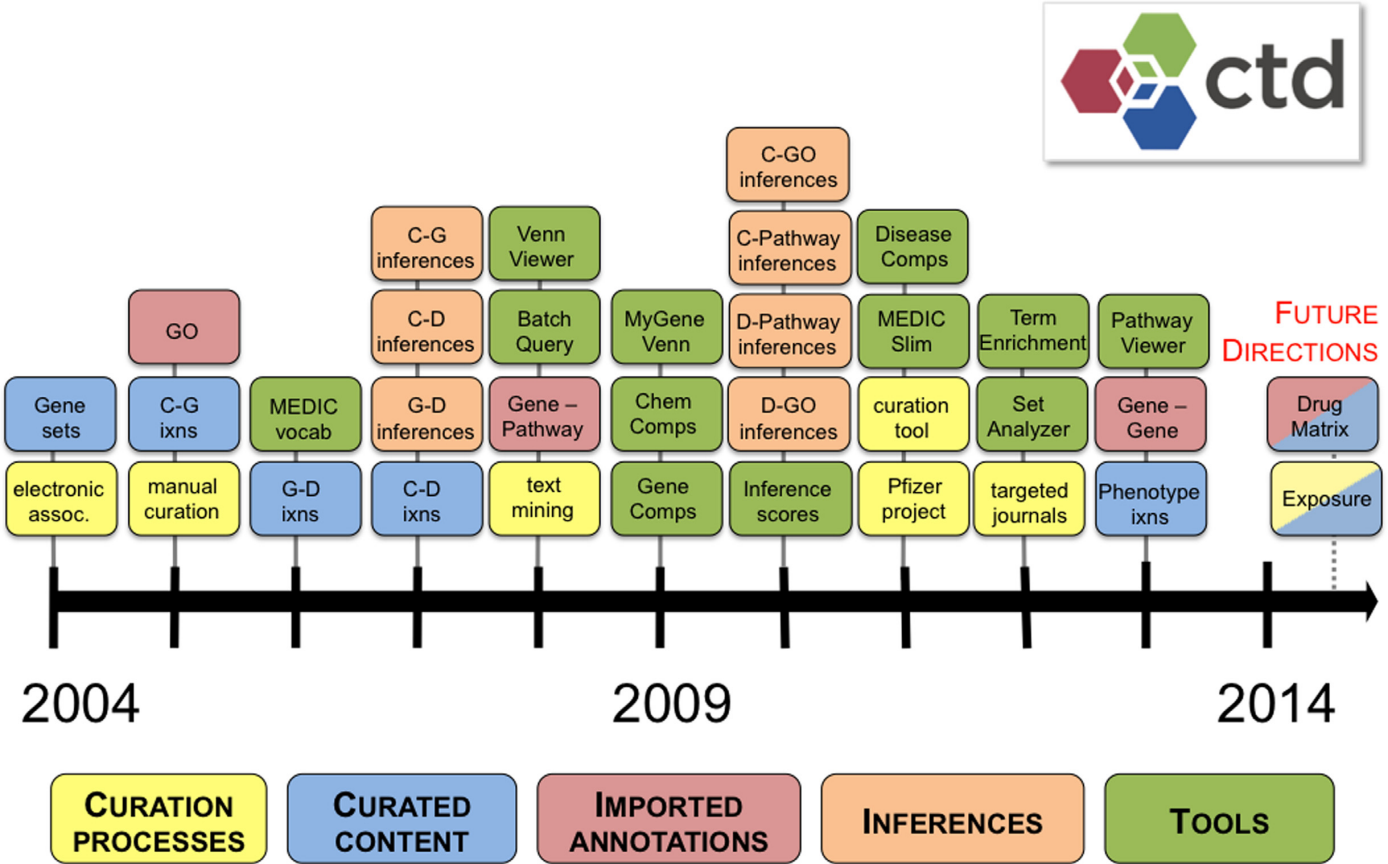
A brief history of CTD. The timeline shows the diversity of CTD's development over the last decade with respect to curation processes (yellow boxes), curated content (blue boxes), imported annotations (red boxes), data inferences (orange boxes) and analytical tools (green boxes). All features have been described in detail in previous CTD publications (http://ctdbase.org/about/publications/#ctdpubs). Abbreviations: C (chemical), G (gene), D (disease), ixns (interactions manually curated from the literature), GO (Gene Ontology), assoc (associations).

Since 2004, CTD has matured primarily in five domains: curation processes, curated content, imported annotations, inference generation and tools to help users explore, visualize and analyze the data (Figure 1). From the beginning, CTD's goal was to promote comparative studies of environmentally important genes across evolutionarily diverse organisms and to integrate them with existing molecular and toxicology resources (2). To accomplish this goal, CTD curated nucleotide and protein sequence data, organized them into cross-species gene sets and leveraged associated PubMed references to search for toxic agents co-mentioned in the titles, abstracts and Medical Subject Headings (MeSH) annotations (1).

The gene sets and electronic associations were eventually replaced with chemical–gene (C–G) interactions that were manually curated from the literature by professional biocurators using controlled vocabularies and structured notation (9). This benefitted CTD in many ways. Instead of relying on co-mentioned terms from an abstract, CTD had Ph.D.-level scientists reading the primary literature and coding the authors’ detailed results in a computable format, increasing the accuracy and reliability of the information (10,11). In 2006, we produced MEDIC (12), a resource of merged OMIM (13) and MeSH (14) disease terms, allowing biocurators to additionally capture chemical–disease (C–D) and gene–disease (G–D) relationships using a robust and hierarchical controlled vocabulary. Controlled vocabularies streamlined the curation process, increased accuracy and consistency and accommodated reproducible query retrievals. Structured notation forced biological events to be represented with a subject and direct object connected by an action term. The appeal and utility of structured notations are evident nowadays with the emergence of other bio-languages, such as BioPax (15) and BEL (http://www.openbel.org/bel-expression-language), whose developers have reached out to CTD to map our interactions into their formats.

In 2007, we implemented an additional layer of integration to generate predicated associations among CTD data, which we call inferences. Specifically, if a chemical has a curated interaction with a gene (C–G) and that same gene has a curated association with a disease (G–D) from another publication, then we establish an inferred relationship between the chemical and the disease (C inferred to D, via G). Inferences provide putative molecular links between otherwise disconnected data to help generate testable hypotheses, transforming knowledge into discoveries. For example, CTD does not currently contain curated data directly linking chemical exposure of bisphenol A and autism; however, CTD does compute a list of 106 inference genes that could possibly connect bisphenol A to autism, based upon curated data. In addition to chemical–gene–disease relationships, inferences also extend to data from GO (16), Kyoto Encyclopedia of Genes and Genomes (KEGG) (17) and Reactome (18) to create a multitude of novel connections. As the number of inferences accrued with increasing manual content, we developed a statistical approach in 2010 to compute ranking scores for each inference to assist with prioritization (19).

While CTD continues to focus on environmental chemicals, in 2010 we collaborated with Pfizer, Inc. to manually curate 88,000 articles describing the toxic actions of pharmaceuticals on cardiovascular, neurological, hepatic and renal systems (20). This project added substantial data for therapeutic compounds.

To accommodate CTD's geometric growth in functionality (and the accompanying resource-intensive processing requirements), we reengineered our technical infrastructure and computational resources in 2007, transforming them into a sophisticated, unified, high-capacity computing environment (9). We continue to successfully leverage and advance this infrastructure today as we further expand upon the content and utility of CTD.

**Figure 2.**
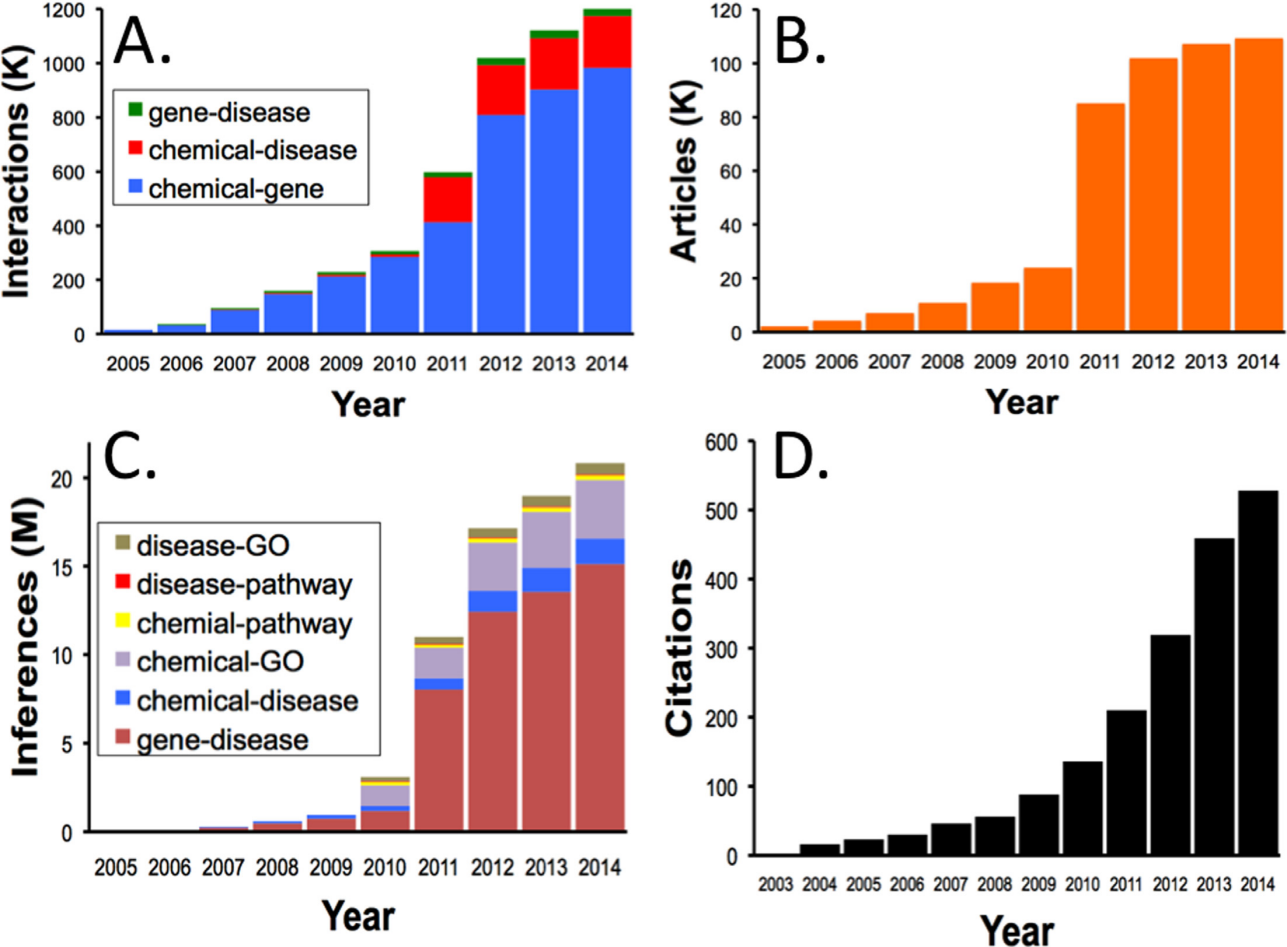
CTD growth. Four graphs show the cumulative growth of CTD over the last 10 years. (**A**) The number of manually curated direct interactions that compose the core triad of chemical–gene, chemical–disease and gene–disease statements (*y* axis in thousands, K). (**B**) The number of manually curated articles from whence the direct interactions in graph A were extracted (*y* axis in thousands, K). (**C**) The number of inferred relationships derived by integrating the direct interactions in graph A with each other as well as with external GO and pathway data sets (*y* axis in millions, M). (**D**) The number of external papers citing their use of CTD. For graphs A, B and C, the content increase in 2011 is our curation derived from the Pfizer project (20). In all four graphs, data for year 2014 are not complete.

Since our beginning, CTD has grown significantly its manually curated content (Figure 2A), the number of curated articles (Figure 2B) and the number of inferences generated by our integration strategies (Figure 2C). Similarly, CTD has been recognized and accepted as a vital resource by the scientific community, being cited in over 500 peer-reviewed publications (Figure 2D) and referenced by public advocacy foundations. In 2013, CTD was seen in the movie *Toxic Hot Seat* (http://www.toxichotseatmovie.com/), a documentary televised on the Home Box Office (HBO) cable network, about the potential health risks associated with flame retardants and fire-related chemicals. Finally, CTD's value is evinced by the more than 50 other databases now presenting our curated data (http://ctdbase.org/about/publications/#use).

## NEW FEATURES

### Increased data content

In July 2014, CTD included 1.2 million manually curated interactions (including 993 361 chemical–gene, 191 592 chemical–disease and 29 605 gene–disease direct interactions) for 13 446 chemicals, 36 393 genes and 6347 diseases extracted from 109 701 peer-reviewed articles (Table 1). Internal integration of these data generated more than 15.2 million inferred gene–disease relationships and 1.4 million inferred chemical–disease relationships. Further integration with external resources including GO annotations and KEGG/Reactome pathways yields additional inferred relationships (Table 1) which are ranked by enrichment for a given chemical or disease. In total, 23.6 million toxicogenomic connections are now provided for analysis and hypothesis development in CTD. When compared against our previous metrics, this reflects a 1.5-fold increase since our 2013 update (8) and a 16-fold increase since our original report in 2009 (6).

**Table 1.** Updated CTD content

Curated data	July 2014
Articles	109 701
Chemicals	13 446
Genes	36 393
Diseases	6347
Relationships	July 2014
Direct chemical–gene interactions	993 361
Direct gene–disease relationships	29 605
Direct chemical–disease relationships	191 592
Inferred gene–disease relationships	15 208 203
Inferred chemical–disease relationships	1 455 061
Enriched chemical–GO relationships	3 360 613
Enriched chemical–pathway relationships	273 063
Inferred disease–pathway relationships	58 099
Gene–GO annotations^a^	1 069 102
Gene–pathway annotations^a^	64 514
Inferred disease–GO relationships	643 814
Gene–gene interactions^a^	284 857
Total relationships	23 631 884

^a^Imported from external databases.

### New toxicogenomic interaction modules

Since pathway analyses provide enhanced information beyond isolated genes and allow researchers to study biological modules, CTD has now integrated gene and protein interaction networks from BioGRID (21). To see what genes/proteins physically interact with their gene of interest, CTD users can select the ‘Gene Interactions’ data tab on any gene page to view, sort and download the experimentally determined interactions. Additionally, CTD's new visualization tool ‘Pathway View’ converts these bi-molecular interactions into a customizable, Cytoscape-based, interactive diagram composed of nodes (genes) and edges (interactions).

**Figure 3.**
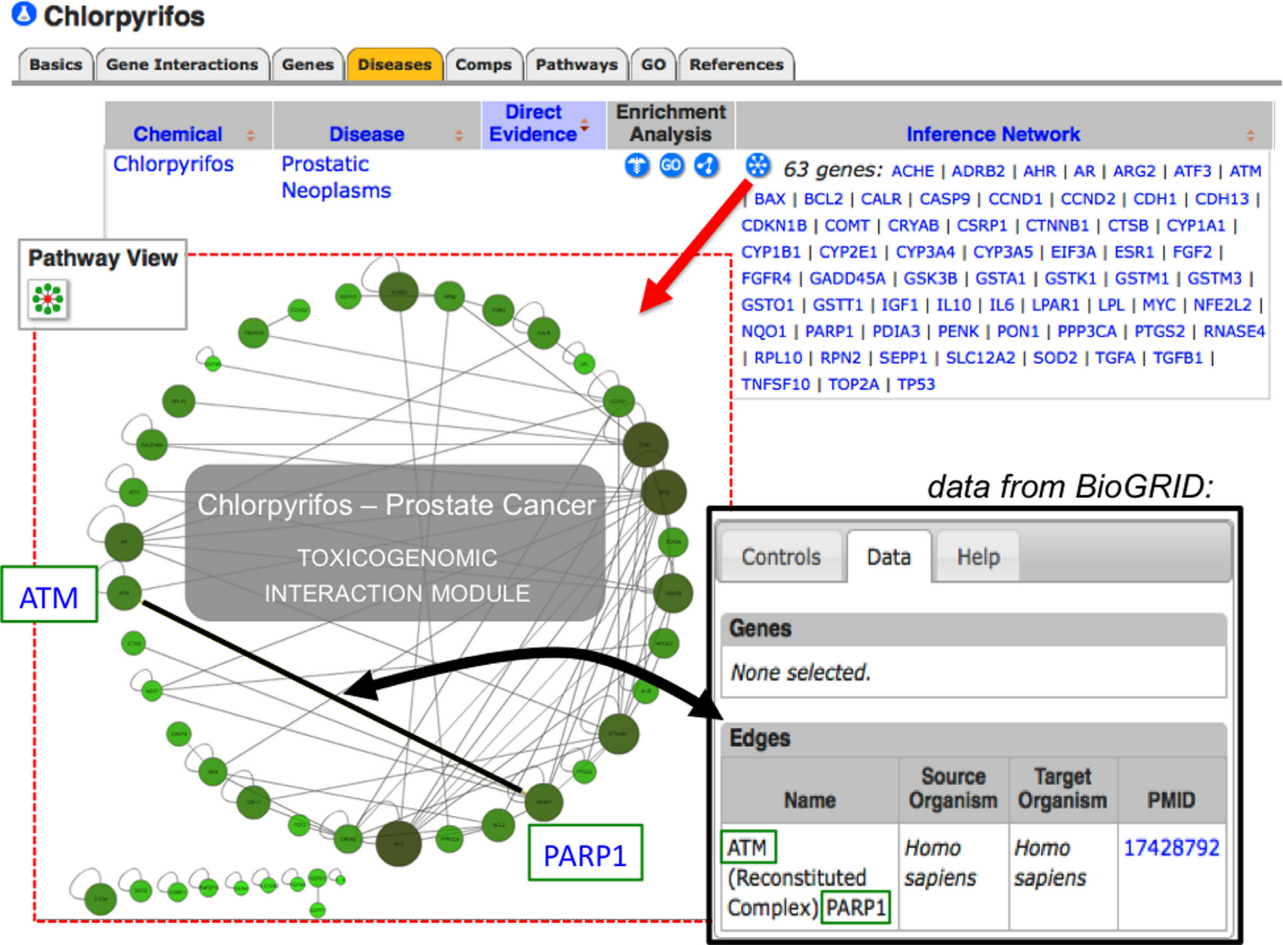
Building potential toxicogenomic network modules from CTD curated content. On the chemical page for chlorpyrifos, the ‘Disease’ data tab (orange) lists the inferred genes that can putatively connect this pesticide to numerous diseases. Here, 63 genes are part of an inference network between chlorpyrifos and prostate cancer, based upon CTD curated content. By clicking on the network icon (red arrow), users launch CTD's new ‘Pathway View’ feature that displays and builds a toxicogenomic interaction module for the inference genes based upon protein and gene interaction data from BioGRID (red dotted inset). The Cytoscape-based map can be easily navigated and customized by the user for a variety of display parameters, as well as be exported to the user's desktop. Edges (black lines) connecting nodes (here, the genes ATM and PARP1) can be clicked to view the BioGRID data tab (black arrow and box) that details the interaction (e.g. the type of assay used, source organism, target organism and PMID reference). The size and color of a gene node is scaled based on the total number of its BioGRID interactions, allowing users to easily detect any highly connected ‘hub’ genes in the network.

Unique to CTD, the gene/protein interactions can be used to build and explore novel toxicogenomic modules for inferred chemical–disease associations based upon CTD curated content. For example, the pesticide chlorpyrifos interacts with 63 genes (C–G) that also have an independent association with the disease prostate cancer (G–D). Thus, chlorpyrifos can be putatively linked to prostate cancer (C inferred to D) by an inference network of 63 genes (Figure 3). The ‘Pathway View’ diagrams are interactive and allow users to explore the experimental details: clicking on edges provides associated experimental details and clicking on nodes provides gene information. The size and color of gene nodes are scaled based on their total number of BioGRID interactions, allowing users to discern any highly connected ‘hub’ genes in the network (19). Our ‘Pathway View’ functionality has also been incorporated in CTD's analytical tool ‘Set Analyzer’ (http://ctdbase.org/tools/analyzer.go), where users can input any list of genes to build an interaction module. This type of meta-analysis builds putative, higher-order biological modules that may help inform toxicological responses.

### New curation practices

Since our last update (8), CTD has adopted two new practices to enhance our curation processes and content:

- *Targeted journal curation*. Keeping a manually curated database both complete and current is a challenging process. To maintain data currency, we implemented targeted journal curation, wherein we target 18 relevant journals for curation upon publication (22). This approach allows us to capture the most current toxicological findings, adding an average of 625 articles each quarter. It is balanced by our complimentary and ongoing chemical-centric curation, which allows us to prioritize chemicals of greatest interest to the general public, research community and regulatory agencies.
- *Fully integrated text mining*. Starting in 2008 (Figure 1), CTD has played an important role in the text-mining community by developing and adapting text-mining strategies to increase curation efficiency and productivity (10,23,24). In 2013, CTD incorporated our text-mining algorithm as part of our standard workflow for chemicals with a large numbers of articles to be curated. This algorithm proved successful in a recent large-scale experiment, wherein it effectively scored and ranked 14 900 articles about heavy metals; this ranking prioritized the literature and resulted in a 27% boost in productivity and a two-fold increase in data content, generating over 41 000 manually curated interactions in just eight weeks (25). Now integrated into our pipeline, text mining is used for abundantly published chemicals to help CTD biocurators focus manual curation efforts on the most relevant articles.

### Phenotypes as a new curated data set

CTD recently started curating chemical–phenotype interactions from the literature. In an initial pilot project (designed to test the curation metrics and feasibility), more than 10 000 articles were reviewed in four months by CTD biocurators to identify pre-disease phenotypes induced by drugs (20). From these articles, over 38 000 interactions were curated using controlled vocabularies, including a subset of MeSH ‘Phenomena and Processes’ terms as an initial phenotype vocabulary. These interactions, while not yet fully integrated within the web-based CTD framework, are freely available to all users as a downloadable Excel spreadsheet (http://ctdbase.org/reports/CTD_pheno_ixns.xls). This file provides the phenotype interactions, PMID article identifiers and the controlled terms connecting 2850 chemicals, 738 genes, 121 phenotypes and 544 anatomical terms for 59 taxa. To our knowledge, this is the first publicly available set of literature-based manually curated chemical–phenotype interactions. The goal is that these interactions will complement high-throughput screening assay data, facilitate cross-species extrapolation via common phenotypes and improve identification of exposure-related effects prior to the onset of disease.

## FUTURE DIRECTIONS

Going forward, we would like to expand our phenotype project with a more versatile vocabulary source, such as the biological process branch of GO, to add greater vertical (granularity) as well as horizontal (broad) coverage of biological concepts.

We also plan to incorporate parts of the DrugMatrix database (26) into CTD (Figure 1), allowing this comprehensive, high-throughput data set to be seen in the context of CTD's curated knowledge. To date, we have imported the DrugMatrix annotations and are manually mapping their terms to our controlled vocabularies to enable the results to be seamlessly integrated with CTD.

Finally, we have developed a new curation process of manually curating the details of real-life exposure studies for integration into CTD. In our exposure curation paradigm, the peer-reviewed literature is manually curated using several controlled vocabularies and free text for over 50 data fields, representing four major knowledge domains defined by the ExO (Exposure Science Ontology): stressor, exposure receptor, event and outcome (27). To date, over 850 articles have been manually curated, resulting in more than 38 000 exposure statements for 646 chemical stressors, 165 exposure receptor populations (from 88 countries), 209 diseases and 119 phenotypic outcomes. Integrating this new data content will help exposure science to be understood and analyzed within CTD's extensive content and visualization tools to find connections to toxicogenomic interactions, GO terms, pathways and gene networks. We hope to begin designing a user-friendly web portal for incorporating exposure data within the CTD framework (Figure 1).

## SUMMARY

CTD was created by and is still managed today by a small team of biologists and software engineers; currently, CTD provides scientists with ∼24 million toxicogenomic connections between chemicals/drugs, genes/proteins and human diseases. Over the decade, it has grown significantly in response to the evolving needs of the toxicology community. Here, on our 10th year anniversary of being on the web, we have reflected upon our progress over the decade, as well as provided our latest updates for increased data content, new toxicogenomic interaction modules, behind-the-scene curation practices that help deliver better and faster content to our users and our new chemical–phenotype interactions.

In recent years, toxicology data have grown exponentially owing largely to high-throughput screening efforts both nationally (28) and internationally (29). This growth has led to the development of several public databases that focus on the actions of chemicals, drugs or small molecules (30, and see U.S. Environmental Protection Agency databases at: http://actor.epa.gov/actor/faces/ACToRHome.jsp). With the exception of emerging zebrafish assays (31), most efforts involve *in vitro* systems, and extrapolation to human toxicity still remains a challenge. CTD's goal is that the depth and diversity of chemicals, model systems and data content in our resource will serve as an important bridge for these other endeavors, and we are working with several groups to ensure that our efforts are complementary wherever possible.

## CITING AND LINKING TO CTD

To cite CTD, please see: http://ctdbase.org/about/publications/#citing. Currently, over 50 external databases link to or present CTD data on their own web sites. If you are interested in establishing links to CTD data, please notify us (http://ctdbase.org/help/contact.go) and follow these instructions: http://ctdbase.org/help/linking.jsp.
